# Glycemic variability and reference percentiles in very low birth weight preterm infants using continuous glucose monitoring

**DOI:** 10.1371/journal.pone.0341593

**Published:** 2026-03-27

**Authors:** Irene Gutiérrez-Rosa, Manuel Lubián-Gutiérrez, Carmen Rodríguez-Barrios, Simón Pedro Lubián-López, Isabel Benavente-Fernández

**Affiliations:** 1 Biomedical Research and Innovation Institute of Cádiz (INiBICA), Puerta del Mar University Hospital, Cádiz, Spain; 2 Division of Neonatology, Department of Paediatrics, Puerta del Mar University Hospital, Cádiz, Spain; 3 Area of Paediatrics, Department of Child and Mother Health and Radiology, Medical School, University of Cádiz, Cádiz, Spain; KI: Karolinska Institutet, SWEDEN

## Abstract

Glycemic control in very-low-birth-weight preterm infants is challenging due to the immaturity of glucose homeostasis during the early postnatal period. The first two weeks of life represent a critical window of metabolic adaptation, characterized by high variability and gradual stabilization of glucose levels; continuous monitoring during this period is therefore essential to capture clinically relevant trends. We aimed to describe daily glucose patterns and variability during the first 14 days and to generate gestational-age–specific reference percentiles for clinically stable preterm infants. A prospective observational study was conducted in a tertiary neonatal intensive care unit (2021–2024), with continuous glucose monitoring during the first 14 days of life. Of 203 eligible infants, 103 clinically stable preterm infants without major comorbidities were included and classified into three gestational age groups (24–26, 27–29, and 30–32 weeks). The median number of glucose readings per infant was 2,557 [1,681–2,959]. Mean glucose concentrations declined over the first two weeks in all groups, with the largest decrease during the initial 72 hours. Infants born at 24–26 weeks had higher mean glucose on day 1 (156.5 ± 47.2 mg/dL) and day 14 (121.4 ± 21.8 mg/dL), and greater variability than those born at 27–29 and 30–32 weeks. Reference percentiles reflected these differences: on day 1, the fifth to ninety-fifth percentiles were 92–245 mg/dL for 24–26 weeks, 65–168 mg/dL for 27–29 weeks, and 67–148 mg/dL for 30–32 weeks; by day 14, they were 73–185, 67–150, and 58–136 mg/dL, respectively.In summary, glucose concentrations and variability decreased with postnatal age, and the least mature infants exhibited higher and more variable values. These gestational-age–specific reference percentiles, derived from continuous monitoring may support individualized glucose management in neonatal intensive care.

## Introduction

Alterations in plasma glucose levels are common in very low birth weight preterm infants (VLBW), especially in those born small for gestational age (SGA) or to mothers with various comorbidities (e.g., diabetes, preeclampsia, etc). Although plasma glucose thresholds defining hypoglycemia (< 47 mg/dL) and hyperglycemia (> 150 mg/dL) in preterm infants are variable, with incidences up to 41% and 81% respectively in the first week of life [1], establishing gestational age (GA)- and day-specific continuous glucose monitoring (CGM) percentiles may assist neonatal intensive care unit (NICU) clinicians in interpreting glycemic values in clinically stable VLBW infants during this early transitional period. These episodes of hypo- or hyperglycaemia have been described up to 2 months of age in clinically stable preterm infants [2].

In VLBW requiring intensive care, where wide fluctuations in blood glucose levels are common, the developing brain is particularly vulnerable to damage from both hypoglycemia and hyperglycemia. Hypoglycaemia has traditionally been associated with brain injury and neurodevelopmental impairment [3], although this association has been questioned in preterm infants [4] and it remains to be determined whether glucose instability causes neuronal injury or is simply a marker of perinatal stress or severity of illness. On the other hand, hyperglycemia has been associated with increased risk of death, retinopathy of prematurity, intraventricular hemorrhage (IVH) and white matter reduction [5].

Current glucose management relies on repeated blood glucose measurements and most studies that have studied the consequences of blood glucose disturbances have been conducted using intermittent blood or plasma glucose measurements, sampled at varying intervals [6]. But in preterm infants it is critical to minimise handling and reduce blood loss, so the use of a less invasive real-time CGM system in these vulnerable infants could allow earlier detection and potentially prevent exposure to extreme glucose levels. Preliminary data suggest that CGM is feasible in preterm infants, shows a strong correlation between CGM and plasma or capillary measurements [7–11], can reduce exposure to prolonged or severe hyperglycaemia and hypoglycaemia [12,13], and is probably cost-effective [14].

CGM is increasingly used in NICUs. Clinically stable preterm infants often display interstitial glucose fluctuations that exceed commonly proposed thresholds for hypo- or hyperglycemia, highlighting the need for easily interpretable, GA- and day-specific reference values to guide clinical interpretation [2]. However, currently available studies have mostly reported pooled mean values across broad postnatal age ranges, included heterogeneous populations, or used intermittent glucose sampling, limiting their applicability to day-to-day bedside care. The absence of GA- and day-specific normative curves makes it difficult to contextualize CGM data and to distinguish physiological adaptation from clinically significant dysglycemia. Establishing such reference curves is therefore essential to identify infants at risk, to support individualized glucose management, and to inform future interventional studies linking glycemic patterns to short- and long-term outcomes.In addition, there is growing recognition that glycemic variability (GV) represents an independent and clinically relevant aspect of neonatal glucose homeostasis. Increased GV has been linked to higher mortality and worse neurodevelopmental outcomes in preterm infants [15,16]. Recent studies show that GV is inversely related to GA—being highest in the most immature neonates—and positively correlated with mean glucose concentrations [16]. Together, these findings underscore the importance of incorporating GV into the interpretation of CGM data in this population.

Therefore, this study aimed to generate dynamic, GA-specific reference curves for glucose concentrations and to characterize GV during the first 2 weeks of life in a cohort of clinically stable VLBW infants without acute comorbidities, since glycemic instability and GA-dependent differences frequently persist beyond the first week and are clinically relevant during this transitional period.

## Methods

### Study design

A prospective, observational cohort study was conducted at a single tertiary-level NICU at Puerta del Mar University Hospital, Cádiz (Spain), between 1 March 2021 and 31 December 2024. The study was approved by the hospital’s Research and Ethics Committee (ref. HUPM-NEO-2020–37, approval date: 25 January 2021). Written informed consent was obtained from parents or legal guardians before enrollment. All data were anonymized prior to analysis, and all procedures were conducted in accordance with the Declaration of Helsinki (2013 revision).

### Study population

All neonates born at ≤ 32 weeks’ GA and with a birth weight ≤ 1500 g, admitted within the first hour of life to the NICU, were consecutively screened for eligibility. Exclusion criteria included chromosomal or genetically confirmed syndromes (usually diagnosed antenatally) and inborn errors of metabolism or other severe congenital metabolic disorders, diagnosed through abnormal newborn screening, metabolic work-up, or genetic confirmation. These criteria were applied prospectively at enrollment, and infants were retrospectively excluded if such diagnoses were made postnatally. Additional exclusions comprised major central nervous system malformations and confirmed congenital infections. To minimize potential confounding factors, we aimed to generate normative, GA- and day-specific reference curves reflecting physiological glucose patterns in clinically stable very preterm infants. Therefore, only infants who (i) had no or grade I germinal matrix hemorrhage (Volpe classification), (ii) remained free of culture-proven sepsis during the first 14 days of life, and (iii) were appropriate for GA at birth were retained for analysis. Sepsis was defined by a positive blood culture or compatible clinical signs with elevated inflammatory markers; episodes were classified as early-onset (≤ 72 h) or late-onset (> 72 h to 14 days). These conditions were excluded because they are well-documented contributors to altered glucose metabolism and greater glycemic variability in preterm infants, which could confound physiological patterns and distort the accuracy of percentile estimation. The cohort was stratified into three GA groups: 24–26 weeks, 27–29 weeks, and 30–32 weeks. Participant recruitment and exclusion processes are summarized in the STROBE flow diagram (S2 Fig), while the overlap between exclusion criteria is illustrated in the Venn diagram (S1 Fig).

### Clinical data collection

Maternal, perinatal, and neonatal data were prospectively recorded, including sex, birth weight Z-score (and small-for-GA status), antenatal steroid exposure, maternal hypertension, gestational diabetes, clinincal and histological chorioamnionitis, mode of delivery and Apgar scores. Operational definitions of maternal and neonatal variables are provided in S13 Table.

### Nutritional management and glucose protocol

Initial stabilization and clinical care followed the European Resuscitation Council and European Consensus Guidelines on the Management of Preterm Infants. Parenteral nutrition was initiated at birth with aminoacids (3 g/kg/day, adjusted as clinically indicated), lipids (1.5 g/kg/day), and glucose (4–6 mg/kg/min), following standardized NICU protocols in alignment with European Society for Paediatric Gastroenterology, Hepatology and Nutrition (ESPGHAN) recommendations. Expressed breast milk (mother’s own or donor) was introduced within the first 24 hours if clinically stable. Parenteral substrates were progressively titrated based on daily biochemical monitoring and clinical tolerance, and discontinued once full enteral feeding was achieved. Hyperglycemia (> 150 mg/dL) was managed with stepwise reductions in the glucose infusion rate (1–2 mg/kg/min, not < 4 mg/kg/min); persistent hyperglycemia > 250 mg/dL for > 3 h prompted intravenous insulin infusion (0.05–0.1 U/kg/h, titrated every 30 min). No infant included in the final cohort required insulin therapy during the 14-day monitoring period. Hypoglycemia (< 47 mg/dL) was treated with a 2 mL/kg bolus of 10% dextrose followed by an increase in the glucose infusion rate. These management strategies were applied uniformly across all GA groups to ensure consistency of metabolic support.

### Continuous glucose monitoring

Glucose dynamics were continuously monitored using the Guardian™ 3 real-time CGM system (Medtronic, Northridge, CA, USA). A 9.5-mm subcutaneous sensor was inserted into the lateral thigh within the first hours of life, transmitting interstitial glucose readings every five minutes to a MiniMed 670G monitor. Calibration was performed at least three times daily using capillary samples measured by a glucose-oxidase analyzer. Discrepancies >15% triggered immediate recalibration. The Guardian™ Sensor 3 used in this study has been previously validated for neonatal use, with studies demonstrating high correlation with plasma glucose values (r ≈ 0.9), minimal bias on Bland–Altman analysis, and >95% of paired measurements within clinically acceptable zones on Clarke and Consensus Error Grids [8,9,11]. Therefore, no additional paired accuracy validation was performed in this cohort. Monitoring continued uninterrupted for 14 days, after which the raw CGM data were exported to a secure REDCap® database.

### Outcomes

The primary outcome was the distribution of interstitial glucose concentrations during the first 14 postnatal days. Secondary outcomes included temporal trends in mean glucose levels and glycaemic variability.

### Statistical methods

All statistical analyses were performed using Stata version 18.0 (StataCorp, College Station, TX, USA). Continuous variables were reported as mean ± standard deviation (SD) or median [interquartile range, IQR], and categorical variables were reported as frequency and percentage. Data normality was assessed using the Shapiro–Wilk test. Group comparisons used χ² or Fisher’s exact test for categorical variables and one-way ANOVA or Kruskal–Wallis tests for continuous variables, as appropriate. When the ANOVA indicated significant differences, pairwise comparisons were conducted using Tukey’s Honest Significant Difference (HSD) test to identify which specific groups differed from each other.

#### Glycaemic reference range estimation.

To define normative glycemic patterns over the first 14 days of life, we used two complementary approaches:

1. Empirical Percentile Estimation: For each GA subgroup (24–26, 27–29, and 30–32 weeks), daily interstitial glucose values were used to calculate the 5th, 10th, 25th, 50th, 75th, 90th, and 95th percentiles, based solely on observed data.
2. Model-Based Percentile Prediction: Linear regression models were fitted to predict specific percentile levels (10th, 50th, 90th) of the glucose distribution as a function of GA (in weeks) and postnatal day (as a continuous variable). Smoothed percentile curves were derived to visualize glucose dynamics and assess alignment with empirical percentiles.

#### Temporal evolution of glucose.

To assess longitudinal trends in glucose concentration over 14 postnatal days:

- A linear mixed-effects model was used to compare mean glucose concentrations between GA groups, incorporating a random intercept for repeated measures. The 24–26 weeks group served as reference.
- One-way ANOVA was performed on each postnatal day to evaluate day-specific group differences, with post-hoc comparisons using Tukey’s HSD test.
- Within-group changes from day 1 to day 14 were assessed using paired t-tests.

#### Glycaemic variability.

GV was assessed using the coefficient of variation (CV), calculated as the ratio of standard deviation to mean glucose concentration over 14 days.

- One-way ANOVA assessed between-group differences in CV, with post-hoc comparisons using Tukey’s HSD test.
- Paired t-tests compared CV between day 1 and day 14 within each group.
- Temporal trends in CV were visualized using LOESS (locally estimated scatterplot smoothing) to generate non-parametric, smoothed curves.

All analyses were conducted using complete-case data.

## Results

### Study population

During the study period, 203 preterm infants (≤32 weeks and/or ≤1500 g) were screened; CGM was placed in 156. After excluding 8 (5.1%) infants for medical reasons or insufficient CGM data, 148 had valid CGM records. Of these, 45 (30.4%) were then excluded based on predefined clinical criteria (16 (10.8%) due to being SGA,18 (12.1%) due to intraventricular hemorrhage (IVH), and 22 (14.8%) due to sepsis) yielding a final cohort of 103 clinically stable infants. Some infants met more than one exclusion criterio (S1 Fig). The inclusion process and final distribution are shown in S2 Fig.

The included population consisted of 103 preterm infants, with a median GA of 30.57 weeks [IQR 28.61–31.71] and a median birth weight of 1400 g [IQR 1090–1540]. Participants were stratified by GA into three groups:13 infants (12.6%) born at 24–26 weeks, 27 infants (26.2%) at 27–29 weeks, and 63 infants (61.2%) at 30–32 weeks of gestation. The distribution of demographic and perinatal characteristics by GA group is summarized in Table 1.

**Table 1:** Perinatal and clinical characteristics of healthy preterm infants stratified by gestational age group. Data are presented as mean ± standard deviation, total number and percentage (%), or median and interquartile range ([IQR]). GA: gestational age.

N (%) or median [IQR]	24-26 GA N = 13 (12.6%)	27-29 GA N = 27 (26.2%)	30-32 GA N = 63 (61.2%)	Total N = 103	p value
Female sex	9 (69.2%)	14 (52%)	29 (46.0%)	52 (50.5%)	0.3092
Birth weight (g)	730 [625-840]	1100 [1000-1250]	1500 [1390-1620]	1400 [1090-1540]	
Caesarean section	7 (53.8%)	17 (63%)	49 (77.8%)	73 (71%)	0.129
Prenatal steroids (partial/complete)	13 (100.0%)	25 (92.6%)	57 (90.5%)	95 (92.2%)	0.865
Magnesium sulphate	11 (84.6%)	19 (70.4%)	39 (61.9%)	69 (67%)	0.285
Prenatal antibiotherapy	11 (84.6%)	12 (44.4%)	30 (47.6%)	53 (51.5%)	0.036*
Clinical chorioamnionitis	3 (23.1%)	5 (18.5%)	7 (11.1%)	15 (14.6%)	0.395
Microbiological chorioamnionitis	1 (7.7%)	1 (3.7%)	2 (3.2%)	4 (3.9%)	0.757
Maternal hypertension	2 (15.4%)	4 (14.8%)	16 (25.4)	22 (21.4%)	0.568
Preeclampsia	2 (15.4%)	4 (14.8%)	18 (28.6%)	24 (23.3%)	0.307
Gestational diabetes	1 (7.7%)	3 (11.1%)	4 (6.3%)	8 (7.8%)	0.664
Apgar 1 min	4 [3-6]	6 [5-7]	8 [6-8]	7 [5-8]	0.001*
Apgar 5 min	6 [6-8]	8 [7-9]	9 [8-9]	8 [7-9]	0.0027*
Days to reach full enteral feeding	10 [7-12]	7 [5-12]	5 [3-7]	6 [4-9]	**0.0001**

*p < 0.05.

### Continuous glucose monitoring data

All included infants underwent CGM during their admission to the neonatal intensive care unit. The median duration of CGM per patient was 250.3 hours [186.2–312.7]. During this period, the median number of glucose measurements per patient was 2557 [IQR: 1681–2959]. Detailed data on CGM duration and the number of glucose measurements stratified by GA are provided in supplementary material S1 Table.

### Percentiles and population sample values

The daily distribution of mean glucose concentrations was analyzed for the entire cohort to describe glycemic trends during the first two weeks of life. In addition to the mean values, 95% confidence intervals (CIs) were calculated to estimate the precision of the population mean for each day. The complete results, including the number of CGM-derived glucose measurements, daily means, and corresponding CIs, are presented in Table 2. Stratified data by GA are available S2–S4 Tables.

**Table 2:** Daily mean glucose concentrations, standard desviation (SD) and 95% confidence intervals (CIs) during the first 14 days of life (n = 103). The table shows the number of glucose measurements, mean glucose concentration, SD and corresponding CIs for each day of life.

Days of life	Number of glucose measurements	Mean (mg/dl)_	SD	CIs
1	13,004	105.43795	±37.36	104.8462 - 106.0297
2	20,103	108.57743	±41.35	108.0445 - 109.1103
3	20,554	106.46051	±28.02	106.1049 - 106.8161
4	20,169	103.89668	±22.02	103.6117 - 104.1816
5	19,164	101.83241	±23.47	101.5234 - 102.1414
6	18,594	101.59054	±28.89	101.205 - 101.9761
7	17,438	101.47533	±31.60	101.0378 - 101.9129
8	15,533	99.393143	±29.01	98.96958 - 99.8167
9	13,074	101.64742	±35.65	101.0862 - 102.2086
10	14,911	99.716438	±32.26	99.22962 - 100.2033
11	13,075	99.737617	±30.92	99.24467 - 100.2306
12	11,121	102.04834	±39.75	101.3662 - 102.7304
13	8,703	98.752846	±29.27	98.18474 - 99.32094
14	5,637	97.520119	±28.80	96.84085 - 98.19939

Predicted percentile values of glucose concentrations were calculated for the entire cohort and separately for each GA group during the first 14 days of life. In infants born at 24–26 weeks of gestation, median glucose values (p50) ranged from 134 mg/dL on day 1–128 mg/dL on day 14. The 5th and 95th percentiles on day 1 were 92 mg/dL and 245 mg/dL, respectively, and 73 mg/dL and 185 mg/dL on day 14. In the 27–29 weeks group, median glucose values (p50) ranged from 100 mg/dL on day 1–99 mg/dL on day 14. The 5th and 95th percentiles on day 1 were 65 mg/dL and 168 mg/dL, respectively, and 67 mg/dL and 150 mg/dL on day 14. In the 30–32 weeks group, median glucose values (p50) ranged from 96 mg/dL on day 1–88 mg/dL on day 14. The 5th and 95th percentiles were 67 mg/dL and 148 mg/dL on day 1, and 58 mg/dL and 136 mg/dL on day 14.

**Fig 1:**
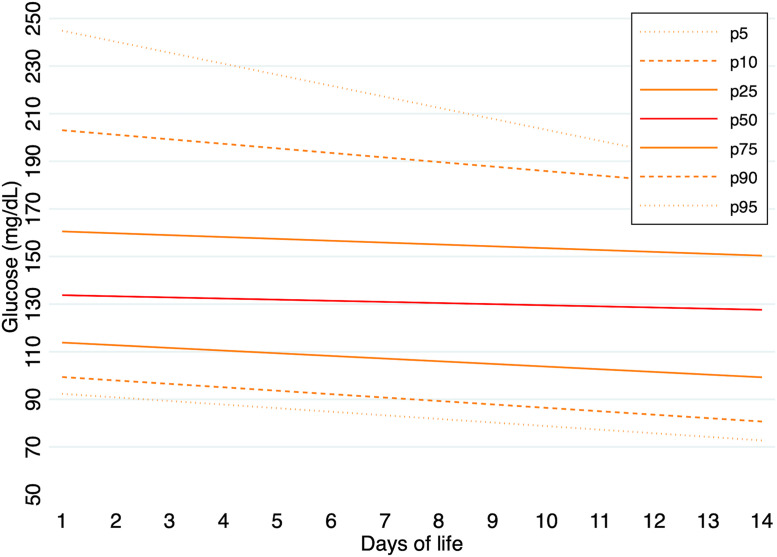
Predicted percentile curves (p10, p25, p50, p75 and p90) of glucose concentrations during the first 14 days of life in infants born at 24–26 weeks of GA.

**Fig 2:**
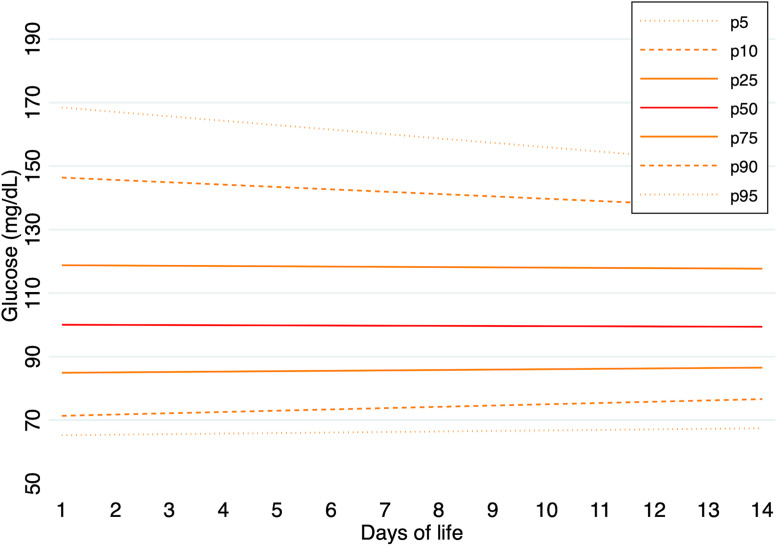
Predicted percentile curves (p10, p25, p50, p75, and p90) of glucose concentrations during the first 14 days of life in infants born at 27–29 weeks of GA.

**Fig 3:**
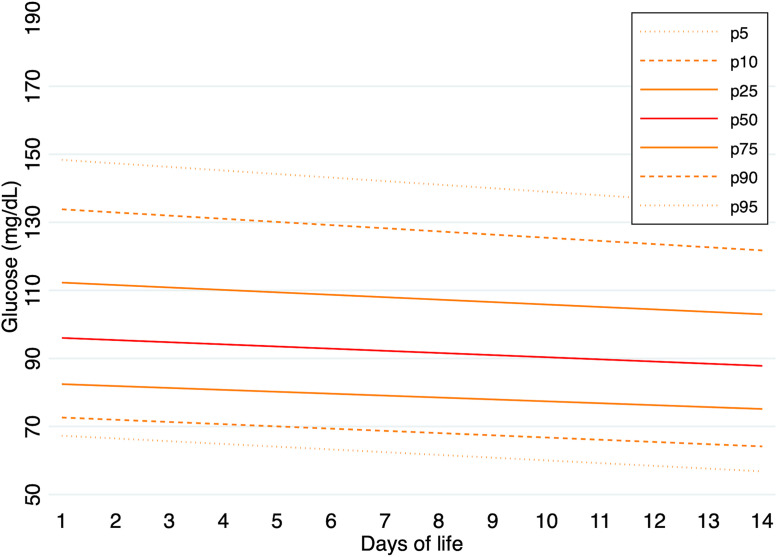
Predicted percentile curves (p10, p25, p50, p75, and p90) of glucose concentrations during the first 14 days of life in infants born at 30–32 weeks of GA.

Predicted percentile values of glucose concentrations, calculated for each GA group during the first 14 days of life, are illustrated in Figs 1–3. A summary of daily 5th and 95th percentile values by GA group is presented in Table 3. The corresponding daily percentile values are provided in S5–S7 Tables.

**Table 3:** Summary of daily glucose predicted percentiles (p5 and p95) by GA group during the first 14 days of life.

Days of life	24-26 GA		27-29 GA		30-32 GA	
	**p5**	**p95**	**p5**	**p95**	**p5**	**p95**
1	92	245	65	168	67	148
2	91	240	65	167	66	147
3	89	236	66	166	66	146
4	88	231	66	164	65	145
5	86	226	66	163	64	144
6	85	222	66	162	63	143
7	83	217	66	160	62	142
8	82	212	66	159	62	141
9	80	208	67	157	61	140
10	79	203	67	156	60	139
11	77	199	67	155	59	138
12	76	194	67	153	58	137
13	74	189	67	152	58	136
14	73	185	67	150	57	135

### Temporal evolution of glucose concentrations and variability

To further explore the overall glycemic behavior in the first 14 days of life the distribution of glucose values was analyzed across the three GA groups (24–26, 27–29, and 30–32 weeks).

**Fig 4:**
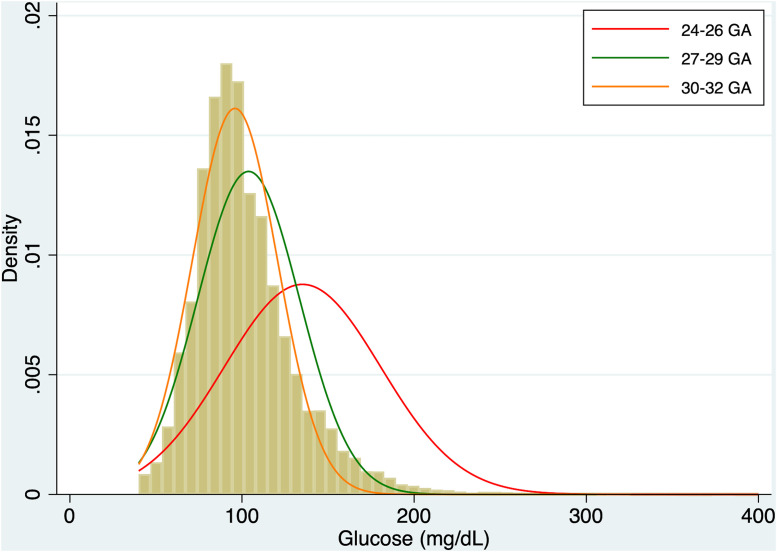
Distribution of glucose concentrations during the first 14 days of life in healthy preterm infants, stratified by GA groups (24–26, 27–29, and 30–32 GA).

The subject-level mean glucose concentrations over the 14 days, differed across groups, with values of 135.4 ± 25.2 mg/dL for the 24–26 weeks GA group, 104.3 ± 15.6 mg/dL for the 27–29 weeks GA group, and 95.6 ± 11.8 mg/dL for the 30–32 weeks GA group. Fig 4 illustrates the kernel density estimation curves for each group, superimposed on the total glucose value histogram.

To address within-subject correlations due to repeated measurements, a linear mixed-effects model with random intercepts was applied using the 24–26 weeks GA group as the reference category. The model yielded an intercept of 135.4 mg/dL (95% CI: 127.3–143.6 mg/dL), corresponding to the mean glucose concentration in the reference group. Compared to this group, glucose concentrations were significantly lower in the 27–29 weeks GA group (estimate: –31.1 mg/dL; 95% CI: –41.0 to –21.2; p < 0.001) and in the 30–32 weeks GA group (estimate: –39.8 mg/dL; 95% CI: –48.8 to –30.9; p < 0.001).

The number of glucose measurements per infant varied among groups, with the 30–32 weeks GA group contributing a higher average number of observations compared to the other groups. These differences in sampling intensity were accounted for in the model, ensuring a robust estimation of group-related differences in glucose levels.

Full results of the linear mixed-effects model, including coefficient estimates, standard errors, z-values, p-values, and 95% confidence intervals, are provided in supplementary material S8 Table.

A detailed day-by-day analysis of glucose concentrations revealed statistically significant differences among GA groups throughout the 14-day observation period. The largest differences were observed during the first days of life. By day 1, the mean glucose concentrations (± standard deviation) were 156.5 ± 47.2 mg/dL in the 24–26 weeks GA group, 105.3 ± 31.5 mg/dL in the 27–29 weeks group, and 94.2 ± 25.6 mg/dL in the 30–32 weeks group (F = 3547.03, p < 0.001). By day 3, the values were 140.6 ± 30.1 mg/dL, 106.4 ± 24.6 mg/dL, and 98.5 ± 22.5 mg/dL, respectively (F = 4010.98, p < 0.001). On day 7, mean glucose concentrations were 123.6 ± 41.1 mg/dL, 109.0 ± 40.0 mg/dL, and 94.1 ± 20.8 mg/dL for the respective groups (F = 1138.50, p < 0.001), and by day 14, the values were 121.4 ± 21.8 mg/dL, 97.2 ± 27.5 mg/dL, and 92.4 ± 28.2 mg/dL (F = 401.67, p < 0.001).

Tukey’s HSD post-hoc testing revealed that all pairwise comparisons were statistically significant (p < 0.05). Infants born at 24–26 weeks of gestation had significantly higher mean glucose levels compared to those born at 27–29 weeks (mean difference: 31.07 mg/dL) and 30–32 weeks (mean difference: 39.81 mg/dL). Additionally, infants born at 27–29 weeks had significantly higher glucose levels than those born at 30–32 weeks (mean difference: 8.7 mg/dL). Detailed post-hoc results are presented in S9 Table.

The 24–26 weeks GA group consistently showed the highest glucose concentrations and the greatest variability, particularly during the first days of life. A general decline in glucose levels was observed over time in all groups, with the most pronounced decrease occurring in the most premature group (from 156.5 mg/dL on day 1 to 121.4 mg/dL on day 14). Standard deviations were also highest on day 1 across all groups, indicating greater glycemic instability during the early postnatal period. Within-group comparisons further confirmed significant temporal changes, with the largest difference observed in the 24–26 weeks GA group (t = 20.96, day 1 vs. day 14; p < 0.001). Despite this decline, statistically significant differences between GA groups persisted through day 14.

**Fig 5:**
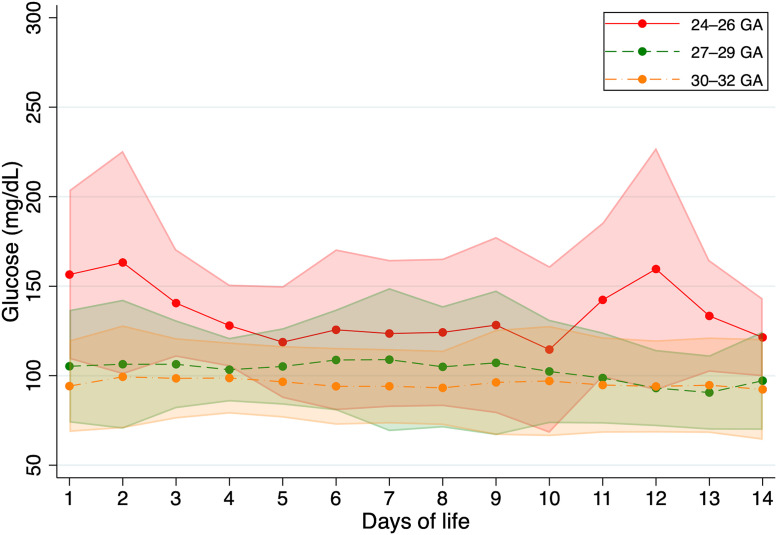
Longitudinal glucose concentration trajectories over the first 14 days of life in healthy preterm infants stratified by gestational age groups (24–26, 27–29, and 30–32 weeks). Solid lines represent the mean glucose concentrations, and shaded areas indicate the standard deviation.

These results, including the temporal evolution of mean glucose concentrations and their corresponding standard deviations across GA groups, are illustrated in Fig 5.

To further characterize the dynamics of glucose regulation, GV was quantified using the CV as a standardized measure of dispersion over the first 14 days of life. The mean CV during the monitoring period was 31.68 ± 8.13% for infants born at 24–26 weeks, 27.81 ± 6.76% for those born at 27–29 weeks, and 25.98 ± 4.28% for the 30–32 weeks group. Although overall glucose variability was comparable across gestational-age strata, the consistency of daily variability patterns improved with advancing maturity, as reflected by progressively lower standard deviations.

When analyzing glucose data within each GA group and subdividing by day of life, distinct temporal and intergroup patterns emerged. Across the observation period, infants born at 24–26 weeks consistently displayed the highest CV values compared to the older gestational groups. The 24–26 weeks group exhibited the greatest day-to-day fluctuation in population GV (SD = 8.13%), whereas the 30–32 weeks group showed the most stable profiles (SD = 4.28%), suggesting maturation of glucose homeostatic mechanisms with increasing GA.

Across the observation period, infants born at 24–26 weeks consistently displayed the highest CV values compared to the older gestational groups. One-way ANOVA revealed a statistically significant difference in CV among GA groups (F(2, 39) = 4.18, p = 0.023). Post-hoc Tukey HSD analysis indicated a significant difference between the 24–26 and 30–32 weeks groups (mean difference = 4.86%, p = 0.020), while the differences between 24–26 and 27–29 weeks (mean difference = 3.33%, p = 0.142) and between 27–29 and 30–32 weeks (mean difference = 1.53%, p = 0.649) were not statistically significant.

Within-group temporal analyses demonstrated significant changes in glucose levels throughout the monitoring period. The 24–26 weeks group exhibited the most pronounced decline from day 1 to day 14 (t = 20.96, p < 0.001), followed by significant decreases in the 27–29 weeks (t = 9.86, p < 0.001) and 30–32 weeks (t = 3.64, p = 0.0003) groups.

**Fig 6:**
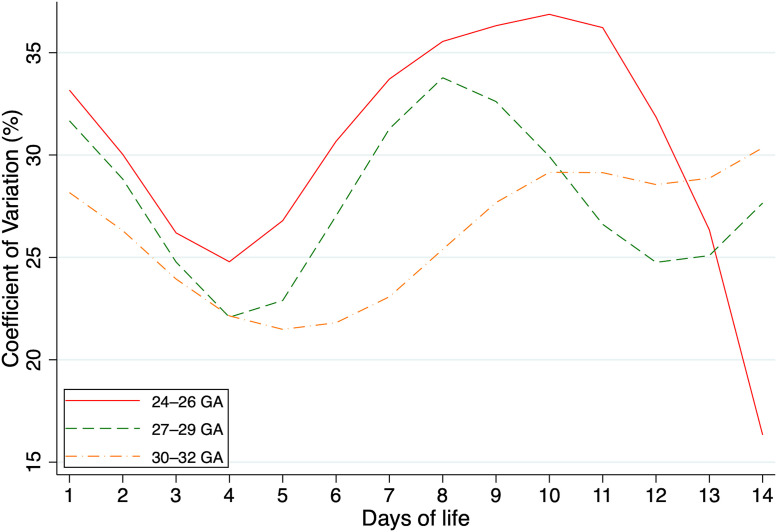
Coefficient of variation of blood glucose levels during the first 14 days of life in preterm infants according to GA groups (24-26, 27-29, and 30-32 weeks). Data were smoothed using LOESS (locally estimated scatterplot smoothing).

The complete daily values of mean glucose, standard deviation, and CV for each GA group are provided in S10–S12 Tables. Temporal evolution of CV is shown in Fig 6.

## Discussion

The present study is the first to provide daily CGM percentile curves (P5–P95) stratified by GA for VLBW infants ≤ 32 weeks’ gestation. Three principal observations emerge. First, a clear GA‑dependent gradient was evident: infants born at 24–26 weeks consistently exhibited higher mean interstitial‑glucose concentrations and greater GV during the initial 72 h than those born at 30–32 weeks, in whom profiles were lower and more stable. Second, although glucose values declined postnatally in all groups, intergroup differences remained significant throughout the 14 day monitoring window. Third, the CV decreased with advancing GA but remained clinically meaningful in the most immature infants even on day 14, underscoring the persistence of metabolic vulnerability beyond the transitional phase.

The absence of the classic early glucose nadir observed in term newborns likely reflects both physiological immaturity and technical limitations. Immature β‑cells with low GLUT‑2 expression restrict insulin release, endogenous hepatic glucose production remains active despite exogenous glucose infusion, peripheral GLUT‑4 density is reduced, and systemic catecholamine‑ and cytokine‑mediated insulin resistance is heightened [17,18]. Consequently, even routine glucose infusion rates may therefore induce hyperglycemia even in otherwise stable preterm infants. From a technical perspective, CGM sensors require a warm‑up and calibration period; preventing data acquisition during the critical first postnatal hour when hypoglycemia is most likely to occur [19]. Altogether, these overlapping factors converge to create persistent hyperglycemia and elevated GV in the most immature infants, underscoring the complex metabolic challenges they face and the need for individualized glucose monitoring and nutritional approaches.

Previous neonatal glycemia studies have largely relied on intermittent plasma sampling in heterogeneous or acutely ill cohorts, limiting their ability to define physiological reference ranges for extremely preterm neonates. Some investigations identified GA‑specific plasma‑glucose nadirs but combined term and preterm infants and captured only the first post‑natal hour [20]. Other studies included at‑risk but otherwise healthy late‑preterm and term infants, again precluding generalization to VLBW populations [21].

Most prior CGM-based investigations in VLBW have focused solely on the first week of life, providing valuable but incomplete data on glucose homeostasis. Some studies demonstrated a high incidence of hyperglycemia and marked variability during this early phase, closely linked to factors such as lower GA and inotrope use, yet they did not explore the persistence of these differences into later postnatal periods [22–24]. Others extended their observations beyond the first month but initiated monitoring only after full enteral nutrition was established, thereby overlooking the critical early transitional patterns [2]. Large multicenter randomized trials have shown that real-time CGM during the first week of life increases the proportion of time within the target glucose range and reduces exposure to prolonged hypo- and hyperglycemia without device-related adverse events. However, these trials were designed to evaluate feasibility and short-term glycemic outcomes, and did not generate GA-specific reference percentiles or investigate the persistence of glycemic differences beyond the early neonatal period [25]. In contrast, the present study fills this gap by enrolling a homogeneous cohort of clinically stable VLBW, explicitly excluding those with complications arising from dysglycemia, and by implementing CGM continuously over the first 14 days of life. This approach enabled the demonstration that GA-dependent differences in both mean glucose levels and GV persist well into the second postnatal week, highlighting the delayed metabolic adaptation in this vulnerable population and providing dynamic percentile references that can inform future individualized care.

The percentile curves generated in this study complement previously published normative CGM data for term infants [26]. However, direct extrapolation of term reference values to very preterm neonates is inappropriate: It has long been argued that neonatal hyperglycemia requires GA-specific definitions [27], and the present data confirm the need for distinct thresholds in the VLBW population. In agreement with earlier observations [22], we observed higher and more variable glucose levels in the least mature infants, but we extended this finding beyond the early transition phase, demonstrating sustained divergence between GA strata up to day 14.

The strengths of our study include a homogeneous cohort of VLBW infants managed with uniform nutritional protocols, the longest CGM duration yet reported in this population (median 246 h), and strict calibration procedures that enhance data integrity. Nevertheless, limitations warrant consideration. First, CGM measures interstitial rather than plasma glucose; during rapid glycemic excursions, notably hypoglycemia, physiological lag can blunt readings [22]. Second, omission of the first postnatal hour means the absolute timing and depth of any initial nadir cannot be ascertained. Third, the 24–26‑week subgroup was relatively small, potentially limiting external validity for the most immature infants. Fourth, current CGM devices are not purpose built for extremely preterm skin, increasing the risk of signal loss or sensor displacement. Fifth, because we deliberately excluded infants with conditions known to affect glucose homeostasis (e.g., SGA, sepsis, IVH ≥ grade II), our reference percentiles are most generalizable to clinically stable VLBW populations rather than the entire spectrum of preterm infants admitted to NICU. Additionally, the limited number of infants with maternal diabetes or postnatal steroid exposure—administered only after day 14 of life—precluded formal subgroup analyses, although these cases did not differ significantly from the main cohort. Finally, although metabolic management was protocolized, unmeasured variations in glucose infusion adjustments could have influenced individual glucose profiles and were not separately analyzed.

Despite these insights, our study did not elucidate the potential causal relationships between early dysglycemia and later clinical outcomes. Additionally, the specific thresholds of glycemia and variability that warrant intervention remain undefined in this population. Further work is needed to determine whether individualized glycemic targets can optimize outcomes beyond glycemic control alone.

The dynamic GA- and day-specific percentiles presented here have several potential clinical applications. They offer a physiologically informed framework to interpret CGM data at the bedside, allowing clinicians to recognize infants whose glucose profiles consistently deviate from expected patterns. Such identification could support timely adjustments in glucose infusion, insulin therapy, or feeding strategies to minimize exposure to harmful extremes of hypo- or hyperglycemia. Beyond clinical monitoring, these reference curves establish a baseline for designing interventional studies aimed at testing whether maintaining glucose levels within GA-specific ranges reduces adverse outcomes. Potential interventions may include fine-tuned glucose infusion protocols, optimization of amino acid and lipid delivery, and early insulin initiation strategies when warranted. Our group is currently investigating associations between glycemic trajectories and major morbidities—including sepsis, necrotizing enterocolitis, severe IVH, and bronchopulmonary dysplasia—as well as long-term neurodevelopmental outcomes. The combination of these longitudinal data with the normative percentiles presented here will inform the design of randomized clinical trials, which are essential to establish causal relationships and to define evidence-based glycemic targets. Only after such trials can standardized, evidence-based clinical protocols be formulated to enable a uniform and evidence-based approach to the management of dysglycemia in neonatal intensive care units.

## Conclusions

Glucose homeostasis in VLBW infants is strongly GA‑dependent. The most immature neonates demonstrated persistently higher and more variable glucose profiles throughout the first two post‑natal weeks. The GA‑specific percentile curves generated in this study constitute a novel reference for the bedside interpretation of CGM data and provide an evidence base for designing interventional trials aimed at optimizing metabolic stability in this vulnerable population.

## Supporting information

S1 Fig Venn diagram illustrating the distribution of 45 excluded patients according to three clinical conditions: small for gestational age (SGA, n = 16), intraventricular hemorrhage (IVH, n = 18), and sepsis within the first 14 days of life (n = 22). Overlapping areas represent patients who met more than one exclusion criterion. Specifically, 4 patients presented both SGA and sepsis, 1 presented both SGA and IVH, and 6 presented both IVH and sepsis. No patients were affected by all three conditions simultaneously. A total of 34 patients had only one of the conditions (SGA: n = 11; IVH: n = 11; sepsis: n = 12).
(PNG)

S2 Fig Flowchart of prospective inclusion and final sample size of the study. GA: weeks of gestational age, VLBWI: very low birth weight infant, CGM: Continuous Glucose Monitoring, IVH: Intraventricular Hemorrhage, SGA: small for gestational age.
(PNG)

S1 Table Median [IQ] continuous glucose monitoring duration and number of glucose values recorded per patient, for the total population and stratified by gestational age group. (DOCX)

S2 Table Daily mean glucose concentrations, standard desviation (SD) and 95% confidence intervals (CIs) during the first 14 days of life in infants born at 24–26 weeks of gestational age (n = 13). The table shows the number of glucose measurements, mean glucose concentration, SD and corresponding CIs for each day of life.
(DOCX)

S3 Table Daily mean glucose concentrations, standard desviation (SD) and 95% confidence intervals (CIs) during the first 14 days of life in infants born at 27–29 weeks of gestational age (n = 27). The table shows the number of glucose measurements, mean glucose concentration, SD and corresponding CIs for each day of life.
(DOCX)

S4 Table Daily mean glucose concentrations, standard desviation (SD) and 95% confidence intervals (CIs) during the first 14 days of life in infants born at 30–32 weeks of gestational age (n = 63). The table shows the number of glucose measurements, mean glucose concentration, SD and corresponding CIs for each day of life.
(DOCX)

S5 Table Predicted percentiles (p5, p10, p25, p50, p75, p90, and p95) of glucose concentrations (mg/dL) by day of life in healthy preterm infants born between 24 and 26 weeks of gestation. (DOCX)

S6 Table Predicted percentiles (P5, P10, P25, P50, P75, P90, and P95) of glucose concentrations (mg/dL) by day of life in healthy preterm infants born between 27 and 29 weeks of gestation. (DOCX)

S7 Table Predicted percentiles (P5, P10, P25, P50, P75, P90, and P95) of glucose concentrations (mg/dL) by day of life in healthy preterm infants born between 30 and 32 weeks of gestation. (DOCX)

S8 Table Results of the linear mixed-effects model assessing the association between gestational age group and mean glucose concentration during the first 14 days of life. The 24–26 weeks gestational age (GA) group was used as the reference category. The model accounts for repeated glucose measurements within individuals by including random intercepts for each subject. Estimates are presented as coefficients with corresponding standard errors, z-values, p-values, and 95% confidence intervals.
(DOCX)

S9 Table Tukey’s Honest Significant Difference (HSD) post-hoc comparisons of mean glucose concentrations between gestational age groups. Values represent mean differences in glucose concentrations (mg/dL) between pairs of gestational age groups, with corresponding 95% confidence intervals and p-values adjusted for multiple comparisons. All pairwise differences were statistically significant (p < 0.05).
(DOCX)

S10 Table Daily glucose concentration data for VLBWI of 24–26 weeks of gestation during the first 14 days of life. The table shows the daily mean glucose values (mg/dL), standard deviations (SD), and corresponding coefficients of variation (CV) as indicators of glycemic variability.
(DOCX)

S11 Table Daily glucose concentration data for VLBWI of 27–29 weeks of gestation during the first 14 days of life. The table shows the daily mean glucose values (mg/dL), standard deviations (SD), and corresponding coefficients of variation (CV) as indicators of glycemic variability.
(DOCX)

S12 Table Daily glucose concentration data for VLBWI of 30–32 weeks of gestation during the first 14 days of life. The table shows the daily mean glucose values (mg/dL), standard deviations (SD), and corresponding coefficients of variation (CV) as indicators of glycemic variability.
(DOCX)

S13 Table Operational definitions of maternal and neonatal variables used in cohort description and analyses. (DOCX)

## References

[1] Alexandrou G, Skiöld B, Karlén J, Tessma MK, Norman M, Adén U, et al. Early hyperglycemia is a risk factor for death and white matter reduction in preterm infants. Pediatrics. 2010;125(3):e584-91. doi: 10.1542/peds.2009-0449 20176674

[2] Mola-Schenzle E, Staffler A, Klemme M, Pellegrini F, Molinaro G, Parhofer KG, et al. Clinically stable very low birthweight infants are at risk for recurrent tissue glucose fluctuations even after fully established enteral nutrition. Arch Dis Child Fetal Neonatal Ed. 2015;100(2):F126-31. doi: 10.1136/archdischild-2014-306168 25381093

[3] Shah R, Harding J, Brown J, McKinlay C. Neonatal Glycaemia and Neurodevelopmental Outcomes: A Systematic Review and Meta-Analysis. Neonatology. 2019;115(2):116–26. doi: 10.1159/000492859 30408811

[4] Goode RH, Rettiganti M, Li J, Lyle RE, Whiteside-Mansell L, Barrett KW, et al. Developmental Outcomes of Preterm Infants With Neonatal Hypoglycemia. Pediatrics. 2016;138(6):e20161424. doi: 10.1542/peds.2016-1424 27940690 PMC5127066

[5] Paulsen ME, Brown SJ, Satrom KM, Scheurer JM, Ramel SE, Rao RB. Long-Term Outcomes after Early Neonatal Hyperglycemia in VLBW Infants: A Systematic Review. Neonatology. 2021;118(5):509–21. doi: 10.1159/000517951 34412051 PMC8530871

[6] Beardsall K. Measurement of glucose levels in the newborn. Early Hum Dev. 2010;86(5):263–7. doi: 10.1016/j.earlhumdev.2010.05.005 20542649

[7] Facchinetti A, Steil GM, Ortiz-rubio P. Continuous glucose monitoring in very preterm infants: a randomized controlled trial. 2017;140(4). 10.1542/peds.2017-1162 28916591

[8] Beardsall K. Real time continuous glucose monitoring in neonatal intensive care. Early Hum Dev. 2019;138:104844. doi: 10.1016/j.earlhumdev.2019.104844 31575451

[9] Beardsall K, Vanhaesebrouck S, Ogilvy-Stuart AL, Vanhole C, VanWeissenbruch M, Midgley P, et al. Validation of the continuous glucose monitoring sensor in preterm infants. Arch Dis Child Fetal Neonatal Ed. 2013;98(2):F136-40. doi: 10.1136/archdischild-2012-301661 22791467

[10] Saw H, Yao N, Chiu C, Chen J. The value of real-time continuous glucose monitoring in premature infants of diabetic mothers. 2017;Dm:4–13. 10.1371/journal.pone.0186486 PMC5643124 29036213

[11] Bonet J, Guiducci S, Res G, Brigadoi S, Sen S, Montaldo P, et al. Continuous Glucose Monitoring among Infants Born Very Preterm: Evidence for Accuracy in Neonatal Intensive Care. J Pediatr. 2025;278:114416. doi: 10.1016/j.jpeds.2024.114416 39579867

[12] Perri A, Giordano L, Corsello M, Priolo F, Vento G, Zecca E, et al. Continuous glucose monitoring (CGM) in very low birth weight newborns needing parenteral nutrition: validation and glycemic percentiles. Ital J Pediatr. 2018;44(1):99. doi: 10.1186/s13052-018-0542-5 30134937 PMC6106728

[13] Beardsall K, Thomson L, Guy C, Bond S, Allison A, Pantaleo B, et al. Continuous glucose monitoring in extremely preterm infants in intensive care: the REACT RCT and pilot study of ‘closed-loop’ technology. Effic Mech Eval. 2021;8:16. doi: 10.3310/eme08160 34723449

[14] Petrou S, Kim S, Bond S, Allison A, Beardsall K, REACT collaborative. Cost-effectiveness of real time continuous glucose monitoring to target glucose control in preterm infants. Semin Perinatol. 2021;45(3):151392. doi: 10.1016/j.semperi.2021.151392 33549333

[15] Fendler W, Walenciak J, Mlynarski W, Piotrowski A. Higher glycemic variability in very low birth weight newborns is associated with greater early neonatal mortality. J Matern Fetal Neonatal Med. 2012;25(7):1122–6. doi: 10.3109/14767058.2011.624220 21923328

[16] Szymońska I, Jagła M, Starzec K, Kwinta P. Glycemic variability in continuous glucose monitoring negatively correlates with gestational age in very low birth weight infants. J Matern Fetal Neonatal Med. 2020;33(17):3041–3. doi: 10.1080/14767058.2019.1566313 30614329

[17] Angelis D, Jaleel MA, Brion LP. Hyperglycemia and prematurity: a narrative review. Pediatr Res. 2023;94(3):892–903. doi: 10.1038/s41390-023-02628-9 37120652

[18] Beardsall K. Hyperglycaemia in the Newborn Infant. Physiology Verses Pathology. Front Pediatr. 2021;9:641306. doi: 10.3389/fped.2021.641306 34368024 PMC8333866

[19] Azevedo N, Del R, Liberatore R, et al. Continuous interstitial glucose monitoring for term newborns: analysis of the first day of life. Published online 2024. doi: 10.1136/archdischild-37580119

[20] Kaiser JR, Bai S, Rozance PJ. Newborn Plasma Glucose Concentration Nadirs by Gestational-Age Group. Neonatology. 2018;113(4):353–9. doi: 10.1159/000487222 29510404

[21] Harris DL, Weston PJ, Harding JE. Incidence of neonatal hypoglycemia in babies identified as at risk. J Pediatr. 2012;161(5):787–91. doi: 10.1016/j.jpeds.2012.05.022 22727868

[22] Beardsall K, Vanhaesebrouck S, Ogilvy-Stuart AL, Vanhole C, Palmer CR, Ong K, et al. Prevalence and determinants of hyperglycemia in very low birth weight infants: cohort analyses of the NIRTURE study. J Pediatr. 2010;157(5):715–9.e1-3. doi: 10.1016/j.jpeds.2010.04.032 20570286

[23] Galderisi A, Facchinetti A, Steil GM, Ortiz-Rubio P, Cavallin F, Tamborlane WV, et al. Continuous Glucose Monitoring in Very Preterm Infants: A Randomized Controlled Trial. Pediatrics. 2017;140(4):e20171162. doi: 10.1542/peds.2017-1162 28916591

[24] Fernández-Martínez MDM, Gómez-Llorente JL, Momblán-Cabo J, Martin-González M, Calvo-Bonachera M, Olvera-Porcel M, et al. Monitoring the incidence, duration and distribution of hyperglycaemia in very-low-birth-weight newborns and identifying associated factors. J Perinat Med. 2020;48(6):631–7. doi: 10.1515/jpm-2020-0074 32432567

[25] Beardsall K, Thomson L, Guy C, Iglesias-Platas I, van Weissenbruch MM, Bond S, et al. Real-time continuous glucose monitoring in preterm infants (REACT): an international, open-label, randomised controlled trial. Lancet Child Adolesc Health. 2021;5(4):265–73. doi: 10.1016/S2352-4642(20)30367-9 33577770 PMC7970623

[26] Harris DL, Weston PJ, Gamble GD, Harding JE. Glucose Profiles in Healthy Term Infants in the First 5 Days: The Glucose in Well Babies (GLOW) Study. J Pediatr. 2020;223:34–41.e4. doi: 10.1016/j.jpeds.2020.02.079 32381469

[27] Decaro MH, Vain NE. Hyperglycaemia in preterm neonates: what to know, what to do. Early Hum Dev. 2011;87 Suppl 1:S19–22. doi: 10.1016/j.earlhumdev.2011.01.005 21276670

