# Supplementary figures and images for: Glycemic variability and reference percentiles in very low birth weight preterm infants using continuous glucose monitoring
Source: PLoS One. 2026 Mar 27;21(3):e0341593. doi: 10.1371/journal.pone.0341593 (PMC13028484; doi:10.1371/journal.pone.0341593)

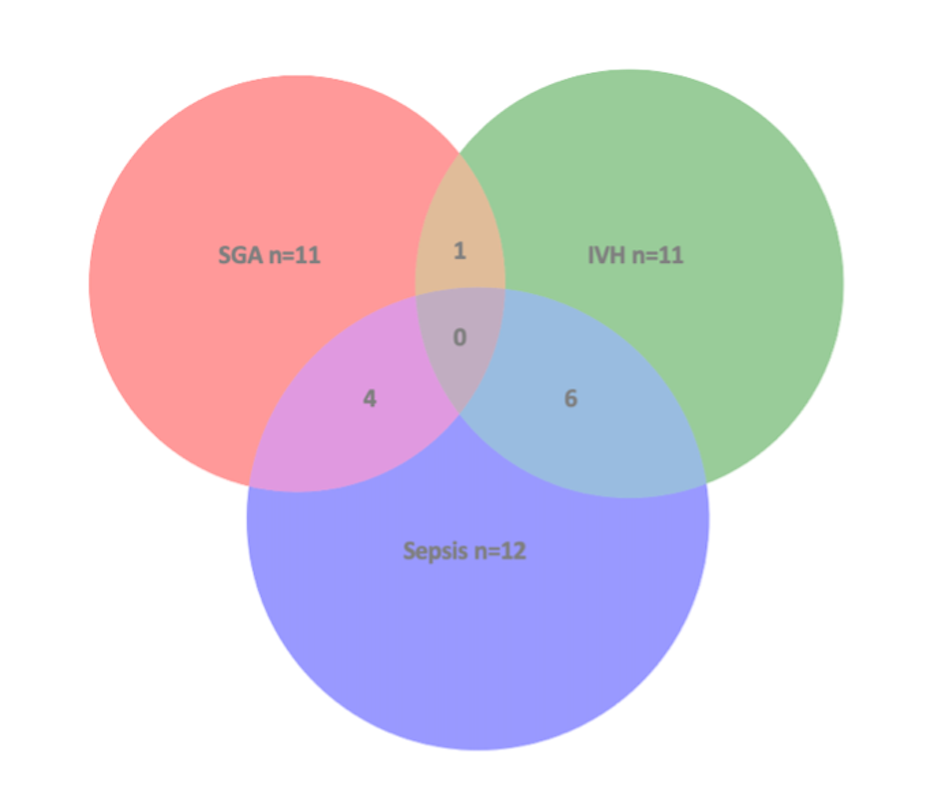

Supplement: S1 Fig — Overlapping areas represent patients who met more than one exclusion criterion. Specifically, 4 patients presented both SGA and sepsis, 1 presented both SGA and IVH, and 6 presented both IVH and sepsis. No patients were affected by all three conditions simultaneously. A total of 34 patients had only one of the conditions (SGA: n = 11; IVH: n = 11; sepsis: n = 12). (PNG) [file pone.0341593.s001.png]

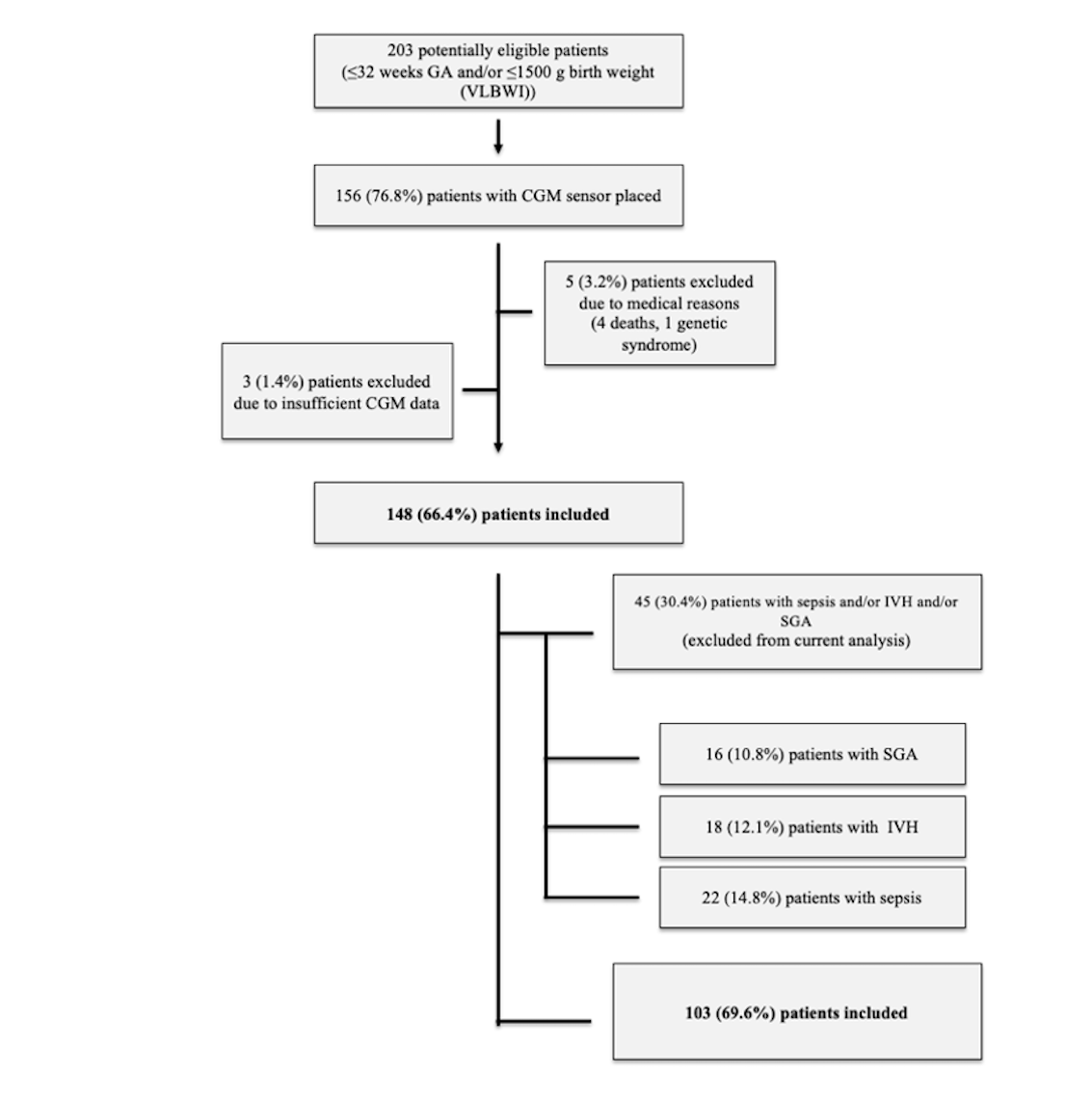

Supplement: S2 Fig — GA: weeks of gestational age, VLBWI: very low birth weight infant, CGM: Continuous Glucose Monitoring, IVH: Intraventricular Hemorrhage, SGA: small for gestational age. (PNG) [file pone.0341593.s002.png]
