# Supplementary material for: Glycemic variability and reference percentiles in very low birth weight preterm infants using continuous glucose monitoring
Source: PLoS One. 2026 Mar 27;21(3):e0341593. doi: 10.1371/journal.pone.0341593 (PMC13028484; doi:10.1371/journal.pone.0341593)
Supplement: S1 Table — (DOCX) [file pone.0341593.s003.docx]

|  | 24–26 GA (n=13) | 27–29 GA (n=27) | 30–32 GA (n=63) | p-value | Total (n=103) |
| --- | --- | --- | --- | --- | --- |
| Monitoring hours (h) | 234.7 [138.5–308.2] | 234.2 [167.3–322.9] | 251.1 [193.3–306.9] | 0.908 | 250.3 [186.2–312.7] |
| Number of glucose values | 2617 [1182–3169] | 2449 [1981–3396] | 2601 [1994–3066] | 0.858 | 2572 [1966–3136] |

**Table S1. Median [IQR] continuous glucose monitoring duration and number of glucose values recorded per patient, for the total population and stratified by gestational age group.**
