# Supplementary material for: Glycemic variability and reference percentiles in very low birth weight preterm infants using continuous glucose monitoring
Source: PLoS One. 2026 Mar 27;21(3):e0341593. doi: 10.1371/journal.pone.0341593 (PMC13028484; doi:10.1371/journal.pone.0341593)
Supplement: S2 Table — The table shows the number of glucose measurements, mean glucose concentration, SD and corresponding CIs for each day of life. (DOCX) [file pone.0341593.s004.docx]

| Days of life | Number of glucose measurements | Mean | SD | CIs |
| --- | --- | --- | --- | --- |
| 1 | 1,845 | 156.50868 | ±47.23 | 154.501 - 158.5164 |
| 2 | 2,458 | 163.22316 | ±62.45 | 160.8826 - 165.5637 |
| 3 | 3,002 | 140.57964 | ±30.08 | 139.5513 - 141.608 |
| 4 | 2,772 | 127.99542 | ±22.90 | 127.1836 - 128.8072 |
| 5 | 2,678 | 118.75885 | ±31.28 | 117.6323 - 119.8855 |
| 6 | 2,038 | 125.65263 | ±44.96 | 123.8245 - 127.4808 |
| 7 | 1,893 | 123.57674 | ±41.11 | 121.8482 - 125.3053 |
| 8 | 1,571 | 124.20349 | ±41.21 | 122.2198 - 126.1872 |
| 9 | 1,120 | 128.27187 | ±49.24 | 125.6534 - 130.8903 |
| 10 | 1,031 | 114.61778 | ±46.58 | 112.0988 - 117.1368 |
| 11 | 949 | 142.34842 | ±43.24 | 139.9361 - 144.7607 |
| 12 | 1,369 | 159.58258 | ±67.81 | 156.3109 - 162.8542 |
| 13 | 1,318 | 133.38896 | ±31.25 | 131.7481 - 135.0299 |
| 14 | 865 | 121.40809 | ±21.80 | 119.9531 - 122.8631 |

**Table S2**. Daily mean glucose concentrations, standard desviation (SD) and 95% confidence intervals (CIs) during the first 14 days of life in infants born at 24–26 weeks of gestational age (n=13). The table shows the number of glucose measurements, mean glucose concentration, SD and corresponding CIs for each day of life.
