# Supplementary material for: Glycemic variability and reference percentiles in very low birth weight preterm infants using continuous glucose monitoring
Source: PLoS One. 2026 Mar 27;21(3):e0341593. doi: 10.1371/journal.pone.0341593 (PMC13028484; doi:10.1371/journal.pone.0341593)
Supplement: S3 Table — The table shows the number of glucose measurements, mean glucose concentration, SD and corresponding CIs for each day of life. (DOCX) [file pone.0341593.s005.docx]

| Days of life | Number of glucose measurements | Mean (mg/dl) | SD | CIs |
| --- | --- | --- | --- | --- |
| 1 | 2,282 | 105.27298 | ±31.48 | 104.2412 - 106.3048 |
| 2 | 3,942 | 106.42429 | ±36.06 | 105.4593 - 107.3893 |
| 3 | 5,081 | 106.39368 | ±24.61 | 105.7945 - 106.9928 |
| 4 | 5,291 | 103.36935 | ±17.79 | 102.9283 - 103.8104 |
| 5 | 4,761 | 105.1633 | ±21.40 | 104.6165 - 105.7101 |
| 6 | 4,989 | 108.78796 | ±28.21 | 108.0755 - 109.5004 |
| 7 | 4,615 | 108.97133 | ±39.98 | 107.9251 - 110.0175 |
| 8 | 3,966 | 104.96674 | ±33.92 | 104.0339 - 105.8996 |
| 9 | 2,390 | 107.19544 | ±40.41 | 105.8826 - 108.5083 |
| 10 | 3,334 | 102.38367 | ±28.92 | 101.5007 - 103.2666 |
| 11 | 3,178 | 98.730743 | ±25.52 | 97.9405 - 99.52098 |
| 12 | 3,080 | 93.108788 | ±21.34 | 92.43593 - 93.78164 |
| 13 | 2,554 | 90.621712 | ±20.81 | 89.89043 - 91.35299 |
| 14 | 1,596 | 97.214386 | ±27.50 | 96.0564 - 98.37237 |

**Table S3.** Daily mean glucose concentrations, standard desviation (SD) and 95% confidence intervals (CIs) during the first 14 days of life in infants born at 27–29 weeks of gestational age (n=27). The table shows the number of glucose measurements, mean glucose concentration, SD and corresponding CIs for each day of life
