# Supplementary material for: Glycemic variability and reference percentiles in very low birth weight preterm infants using continuous glucose monitoring
Source: PLoS One. 2026 Mar 27;21(3):e0341593. doi: 10.1371/journal.pone.0341593 (PMC13028484; doi:10.1371/journal.pone.0341593)
Supplement: S4 Table — The table shows the number of glucose measurements, mean glucose concentration, SD and corresponding CIs for each day of life. (DOCX) [file pone.0341593.s006.docx]

| Days of life | Number of glucose measurements | Mean (mg/dl) | SD | CIs |
| --- | --- | --- | --- | --- |
| 1 | 8,877 | 94.186348 | ±25.64 | 93.67357 - 94.69913 |
| 2 | 13,703 | 99.391815 | ±28.76 | 98.93188 - 99.85176 |
| 3 | 12,471 | 98.52562 | ±22.49 | 98.15424 - 98.897 |
| 4 | 12,106 | 98.731873 | ±19.91 | 98.39765 - 99.06609 |
| 5 | 11,725 | 96.594612 | ±20.03 | 96.2545 - 96.93473 |
| 6 | 11,567 | 94.080734 | ±21.50 | 93.71419 - 94.44728 |
| 7 | 10,930 | 94.113472 | ±20.83 | 93.74445 - 94.48249 |
| 8 | 9,996 | 93.229233 | ±20.82 | 92.84485 - 93.61362 |
| 9 | 9,564 | 96.275055 | ±29.42 | 95.71239 - 96.83772 |
| 10 | 10,546 | 97.039352 | ±30.82 | 96.47442 - 97.60428 |
| 11 | 8,948 | 94.806908 | ±26.70 | 94.27994 - 95.33388 |
| 12 | 6,672 | 94.012749 | ±25.77 | 93.43048 - 94.59502 |
| 13 | 4,831 | 94.707939 | ±26.70 | 94.01417 - 95.40171 |
| 14 | 3,176 | 92.357513 | ±28.20 | 91.46899 - 93.24603 |

**Table S4**. Daily mean glucose concentrations, standard desviation (SD) and 95% confidence intervals (CIs) during the first 14 days of life in infants born at 30–32 weeks of gestational age (n=63). The table shows the number of glucose measurements, mean glucose concentration, SD and corresponding CIs for each day of life.
