# Supplementary material for: Glycemic variability and reference percentiles in very low birth weight preterm infants using continuous glucose monitoring
Source: PLoS One. 2026 Mar 27;21(3):e0341593. doi: 10.1371/journal.pone.0341593 (PMC13028484; doi:10.1371/journal.pone.0341593)
Supplement: S5 Table — (DOCX) [file pone.0341593.s007.docx]

| Days of life | p5 | p10 | p25 | p50 | p75 | p90 | p95 |
| --- | --- | --- | --- | --- | --- | --- | --- |
| 1 | 92 | 99 | 114 | 134 | 161 | 203 | 245 |
| 2 | 91 | 98 | 113 | 133 | 160 | 201 | 240 |
| 3 | 89 | 96 | 112 | 133 | 159 | 199 | 236 |
| 4 | 88 | 95 | 110 | 132 | 158 | 197 | 231 |
| 5 | 86 | 94 | 109 | 132 | 157 | 195 | 226 |
| 6 | 85 | 92 | 108 | 131 | 157 | 194 | 222 |
| 7 | 83 | 91 | 107 | 131 | 156 | 192 | 217 |
| 8 | 82 | 89 | 106 | 130 | 155 | 190 | 212 |
| 9 | 80 | 88 | 105 | 130 | 154 | 188 | 208 |
| 10 | 79 | 86 | 104 | 130 | 154 | 186 | 203 |
| 11 | 77 | 85 | 103 | 129 | 153 | 184 | 199 |
| 12 | 76 | 84 | 102 | 129 | 152 | 182 | 194 |
| 13 | 74 | 82 | 100 | 128 | 151 | 180 | 189 |
| 14 | 73 | 81 | 99 | 128 | 150 | 178 | 185 |

**Table S5.** Predicted percentiles (p5, p10, p25, p50, p75, p90, and p95) of glucose concentrations (mg/dL) by day of life in healthy preterm infants born between 24 and 26 weeks of gestation.
