# Supplementary material for: Glycemic variability and reference percentiles in very low birth weight preterm infants using continuous glucose monitoring
Source: PLoS One. 2026 Mar 27;21(3):e0341593. doi: 10.1371/journal.pone.0341593 (PMC13028484; doi:10.1371/journal.pone.0341593)
Supplement: S6 Table — (DOCX) [file pone.0341593.s008.docx]

| Days of life | p5 | p10 | p25 | p50 | p75 | p90 | p95 |
| --- | --- | --- | --- | --- | --- | --- | --- |
| 1 | 65 | 71 | 85 | 100 | 119 | 146 | 168 |
| 2 | 65 | 72 | 85 | 100 | 119 | 146 | 167 |
| 3 | 66 | 72 | 85 | 100 | 119 | 145 | 166 |
| 4 | 66 | 73 | 85 | 100 | 119 | 144 | 164 |
| 5 | 66 | 73 | 85 | 100 | 118 | 143 | 163 |
| 6 | 66 | 73 | 86 | 100 | 118 | 143 | 162 |
| 7 | 66 | 74 | 86 | 100 | 118 | 142 | 160 |
| 8 | 66 | 74 | 86 | 100 | 118 | 141 | 159 |
| 9 | 67 | 75 | 86 | 100 | 118 | 140 | 157 |
| 10 | 67 | 75 | 86 | 100 | 118 | 140 | 156 |
| 11 | 67 | 75 | 86 | 100 | 118 | 139 | 155 |
| 12 | 67 | 76 | 86 | 100 | 118 | 138 | 153 |
| 13 | 67 | 76 | 86 | 99 | 118 | 138 | 152 |
| 14 | 67 | 77 | 87 | 99 | 118 | 137 | 150 |

**Table S6.**  Predicted percentiles (P5, P10, P25, P50, P75, P90, and P95) of glucose concentrations (mg/dL) by day of life in healthy preterm infants born between 27 and 29 weeks of gestation
