# Supplementary material for: Glycemic variability and reference percentiles in very low birth weight preterm infants using continuous glucose monitoring
Source: PLoS One. 2026 Mar 27;21(3):e0341593. doi: 10.1371/journal.pone.0341593 (PMC13028484; doi:10.1371/journal.pone.0341593)
Supplement: S7 Table — (DOCX) [file pone.0341593.s009.docx]

| Days of life | p5 | p10 | p25 | p50 | p75 | p90 | p95 |
| --- | --- | --- | --- | --- | --- | --- | --- |
| 1 | 67 | 73 | 82 | 96 | 112 | 134 | 148 |
| 2 | 66 | 72 | 82 | 95 | 112 | 133 | 147 |
| 3 | 66 | 71 | 81 | 95 | 111 | 132 | 146 |
| 4 | 65 | 71 | 81 | 94 | 110 | 131 | 145 |
| 5 | 64 | 70 | 80 | 94 | 109 | 130 | 144 |
| 6 | 63 | 69 | 80 | 93 | 109 | 129 | 143 |
| 7 | 62 | 69 | 79 | 92 | 108 | 128 | 142 |
| 8 | 62 | 68 | 79 | 92 | 107 | 127 | 141 |
| 9 | 61 | 67 | 78 | 91 | 107 | 126 | 140 |
| 10 | 60 | 67 | 77 | 90 | 106 | 125 | 139 |
| 11 | 59 | 66 | 77 | 90 | 105 | 125 | 138 |
| 12 | 58 | 65 | 76 | 89 | 104 | 124 | 137 |
| 13 | 58 | 65 | 76 | 88 | 104 | 123 | 136 |
| 14 | 57 | 64 | 75 | 88 | 103 | 122 | 135 |

**Table S7.** Predicted percentiles (P5, P10, P25, P50, P75, P90, and P95) of glucose concentrations (mg/dL) by day of life in healthy preterm infants born between 30 and 32 weeks of gestation.
