# Supplementary material for: Glycemic variability and reference percentiles in very low birth weight preterm infants using continuous glucose monitoring
Source: PLoS One. 2026 Mar 27;21(3):e0341593. doi: 10.1371/journal.pone.0341593 (PMC13028484; doi:10.1371/journal.pone.0341593)

**Table S8.** Results of the linear mixed-effects model assessing the association between gestational age group and mean glucose concentration during the first 14 days of life.
The 24–26 weeks gestational age (GA) group was used as the reference category. The model accounts for repeated glucose measurements within individuals by including random intercepts for each subject. Estimates are presented as coefficients with corresponding standard errors, z-values, p-values, and 95% confidence intervals.


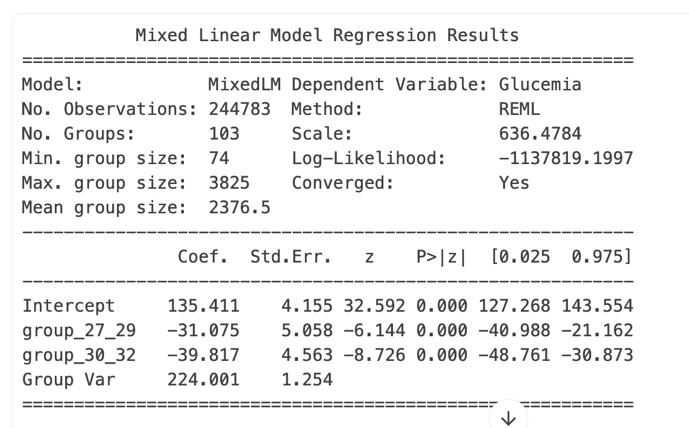

Supplement: S8 Table — The 24–26 weeks gestational age (GA) group was used as the reference category. The model accounts for repeated glucose measurements within individuals by including random intercepts for each subject. Estimates are presented as coefficients with corresponding standard errors, z-values, p-values, and 95% confidence intervals. (DOCX) [file pone.0341593.s010.docx]
