# Supplementary material for: Glycemic variability and reference percentiles in very low birth weight preterm infants using continuous glucose monitoring
Source: PLoS One. 2026 Mar 27;21(3):e0341593. doi: 10.1371/journal.pone.0341593 (PMC13028484; doi:10.1371/journal.pone.0341593)
Supplement: S9 Table — Values represent mean differences in glucose concentrations (mg/dL) between pairs of gestational age groups, with corresponding 95% confidence intervals and p-values adjusted for multiple comparisons. All pairwise differences were statistically significant (p < 0.05). (DOCX) [file pone.0341593.s011.docx]

| **Comparison (GA groups)** | **Mean difference (mg/dL)** | **Std. Error** | **t value** | **p-value** | **95% Confidence Interval** |
| --- | --- | --- | --- | --- | --- |
| **27–29 GA vs 24–26 GA** | -31.079 | 5.063 | -6.138 | <0.001 | [-43.124 -19.033] |
| **30–32 GA vs 24–26 GA** | -39.815 | 4.569 | -8.715 | <0.001 | [-50.685 -28.946] |
| **30–32 GA vs 27–29 GA** | -8.736 | 3.45 | -2.532 | 0.0342 | [-16.944 -0.529] |

**Table S9**. Tukey’s Honest Significant Difference (HSD) post-hoc comparisons of mean glucose concentrations between gestational age groups.Values represent mean differences in glucose concentrations (mg/dL) between pairs of gestational age groups, with corresponding 95% confidence intervals and p-values adjusted for multiple comparisons. All pairwise differences were statistically significant (p < 0.05).
