# Supplementary material for: Glycemic variability and reference percentiles in very low birth weight preterm infants using continuous glucose monitoring
Source: PLoS One. 2026 Mar 27;21(3):e0341593. doi: 10.1371/journal.pone.0341593 (PMC13028484; doi:10.1371/journal.pone.0341593)
Supplement: S10 Table — The table shows the daily mean glucose values (mg/dL), standard deviations (SD), and corresponding coefficients of variation (CV) as indicators of glycemic variability. (DOCX) [file pone.0341593.s012.docx]

| Days of life | Mean | SD | CV |
| --- | --- | --- | --- |
| 1 | 156.51 | 47.24 | 30.18 |
| 2 | 163.22 | 62.46 | 38.27 |
| 3 | 140.58 | 30.08 | 21.40 |
| 4 | 128.00 | 22.90 | 17.89 |
| 5 | 118.76 | 31.29 | 26.34 |
| 6 | 125.65 | 44.96 | 35.78 |
| 7 | 123.58 | 41.12 | 33.27 |
| 8 | 124.20 | 41.22 | 33.19 |
| 9 | 128.27 | 49.24 | 38.39 |
| 10 | 114.62 | 46.58 | 40.64 |
| 11 | 142.35 | 43.25 | 30.38 |
| 12 | 159.58 | 67.82 | 42.50 |
| 13 | 133.39 | 31.25 | 23.43 |
| 14 | 121.41 | 21.80 | 17.96 |

**Table S10.** Daily glucose concentration data for VLBWI of 24–26 weeks of gestation during the first 14 days of life. The table shows the daily mean glucose values (mg/dL), standard deviations (SD), and corresponding coefficients of variation (CV) as indicators of glycemic variability.
