# Supplementary material for: Glycemic variability and reference percentiles in very low birth weight preterm infants using continuous glucose monitoring
Source: PLoS One. 2026 Mar 27;21(3):e0341593. doi: 10.1371/journal.pone.0341593 (PMC13028484; doi:10.1371/journal.pone.0341593)
Supplement: S11 Table — The table shows the daily mean glucose values (mg/dL), standard deviations (SD), and corresponding coefficients of variation (CV) as indicators of glycemic variability. (DOCX) [file pone.0341593.s013.docx]

| Days of life | Mean | SD | CV |
| --- | --- | --- | --- |
| 1 | 105.27 | 31.48 | 29.91 |
| 2 | 106.42 | 36.07 | 33.89 |
| 3 | 106.39 | 24.61 | 23.13 |
| 4 | 103.37 | 17.80 | 17.22 |
| 5 | 105.16 | 21.41 | 20.36 |
| 6 | 108.79 | 28.22 | 25.94 |
| 7 | 108.97 | 39.99 | 36.70 |
| 8 | 104.97 | 33.92 | 32.32 |
| 9 | 107.20 | 40.41 | 37.70 |
| 10 | 102.38 | 28.93 | 28.26 |
| 11 | 98.73 | 25.53 | 25.86 |
| 12 | 93.11 | 21.35 | 22.93 |
| 13 | 90.62 | 20.81 | 22.97 |
| 14 | 97.21 | 27.50 | 28.29 |

**Table S11.** Daily glucose concentration data for VLBWI of 27–29 weeks of gestation during the first 14 days of life. The table shows the daily mean glucose values (mg/dL), standard deviations (SD), and corresponding coefficients of variation (CV) as indicators of glycemic variability.
