# Supplementary material for: Glycemic variability and reference percentiles in very low birth weight preterm infants using continuous glucose monitoring
Source: PLoS One. 2026 Mar 27;21(3):e0341593. doi: 10.1371/journal.pone.0341593 (PMC13028484; doi:10.1371/journal.pone.0341593)
Supplement: S12 Table — The table shows the daily mean glucose values (mg/dL), standard deviations (SD), and corresponding coefficients of variation (CV) as indicators of glycemic variability. (DOCX) [file pone.0341593.s014.docx]

| Days of life | Mean | SD | CV | |
| --- | --- | --- | --- | --- |
| 1 | 94.19 | 25.65 | 27.23 | |
| 2 | 99.39 | 28.77 | 28.94 | |
| 3 | 98.53 | 22.49 | 22.83 | |
| 4 | 98.73 | 19.91 | 20.17 | |
| 5 | 96.59 | 20.03 | 20.74 | |
| 6 | 94.08 | 21.51 | 22.86 | |
| 7 | 94.11 | 20.84 | 22.14 | |
| 8 | 93.23 | 20.83 | 22.34 | |
| 9 | 96.28 | 29.42 | 30.56 | |
| 10 | 97.04 | 30.82 | 31.76 | |
| 11 | 94.81 | 26.71 | 28.17 | |
| 12 | 94.01 | 25.78 | 27.42 | |
| 13 | 94.71 | 26.70 | 28.20 | |
| 14 | 92.36 | 28.21 | | 30.54 |

**Table S12.** Daily glucose concentration data for VLBWI of 30–32 weeks of gestation during the first 14 days of life. The table shows the daily mean glucose values (mg/dL), standard deviations (SD), and corresponding coefficients of variation (CV) as indicators of glycemic variability.
