# Supplementary material for: Glycemic variability and reference percentiles in very low birth weight preterm infants using continuous glucose monitoring
Source: PLoS One. 2026 Mar 27;21(3):e0341593. doi: 10.1371/journal.pone.0341593 (PMC13028484; doi:10.1371/journal.pone.0341593)
Supplement: S13 Table — (DOCX) [file pone.0341593.s015.docx]

| Variable | Definition |
| --- | --- |
| Maternal hypertension | Systolic BP ≥140 mmHg or diastolic BP ≥90 mmHg after 20 weeks of gestation on ≥2 occasions |
| Preeclampsia | Maternal hypertension plus proteinuria (≥300 mg/24h) or signs of end-organ dysfunction |
| Gestational diabetes | Glucose intolerance first recognized during pregnancy, diagnosed by screening with an O’Sullivan test followed by a 100-g or 75-g oral glucose tolerance test according to local protocol, in the absence of criteria for overt diabetes in the first trimester (fasting glucose ≥126 mg/dL, HbA1c ≥6.5%, or random glucose ≥200 mg/dL). |
| Clinical chorioamnionitis | \|  \| \| --- \|  \| Diagnosis based on ≥1 of the following: maternal fever ≥38°C, uterine tenderness, maternal or fetal tachycardia, foul/purulent amniotic fluid, leukocytosis >15,000/µL \| \| --- \| |
| Microbiological chorioamnionitis | Positive amniotic fluid culture, placental culture, or histopathological confirmation of acute chorioamnionitis |
| Prenatal antibiotherapy | Administration of intravenous or intramuscular antibiotics to the mother within 72 h before delivery, typically for prolonged rupture of membranes, chorioamnionitis, or intrapartum GBS prophylaxis |
| Early-onset sepsis | Positive blood culture or clinical signs with elevated inflammatory markers ≤72h of life |
| Late-onset sepsis | Positive blood culture or clinical signs with elevated inflammatory markers >72h to day 14 of life |
| Small for gestational age (SGA) | Birth weight <10th percentile for gestational age using Fenton growth charts. |
| Intraventricular hemorrhage (IVH) | Volpe classification (cranial ultrasound): **Grade I:** Subependymal germinal matrix hemorrhage; **Grade II:** IVH filling <50% of ventricular area without ventricular dilation; **Grade III:** IVH filling >50% with acute ventricular dilatation. **Periventricular hemorrhagic infarction (PVHI):** venous infarction with parenchymal involvement |

**Table S13.** Operational definitions of maternal and neonatal variables used in cohort description and analyses.
